# Efficient Expression of Xylanase by Codon Optimization and Its Effects on the Growth Performance and Carcass Characteristics of Broiler

**DOI:** 10.3390/ani9020065

**Published:** 2019-02-20

**Authors:** Hong Hu, Sifa Dai, Aiyou Wen, Xi Bai

**Affiliations:** College of Animal Science, Anhui Science and Technology University, Fengyang 233100, China; haiyanghh@163.com (H.H.); daisifa-004@163.com (S.D.); aywen2008@126.com (A.W.)

**Keywords:** xylanase, codon optimization, expression, *Pichia pastoris*, broiler, growth performance, carcass characteristics

## Abstract

**Simple Summary:**

The aim of this work was to combine xylanase expression and broiler production. The xylanase (*XynB*) gene from *Trichoderma reesei* was optimized to increase its expression level in *Pichia pastoris*. The maximum activity of xylanase (optiXynB) secreted by *P. pastoris* pPICZaA-optiXynB was 1299 U/mL after 96 h induction. The recombinase was highly specific towards birchwood xylan, beechwood xylan, and oat-spelt xylan. Dietary 1000 and 1500 IU/kg optiXynB significantly increased (*p* < 0.05) final weight and body weight gain; dietary 500, 1000, and 1500 IU/kg optiXynB significantly increased (*p* < 0.05) pre-evisceration weight, dressed percentage, and eviscerated weight compared with the control group. Results suggested that the optiXynB from *P. pastoris* pPICZaA-optiXynB has great application in broiler production.

**Abstract:**

The aim of the present study was to improve the expression level of *Trichoderma reesei* xylanase (XynB) in *Pichia pastoris* through a codon optimization strategy and evaluate its effects on the growth performance and carcass characteristics of broiler. According to the codon bias of *Pichia* genome, the *XynB* gene from *T. reesei* was optimized and synthesized by whole gene assembly to improve its expression level in *P. pastoris*. Approximately 180 target mutations were successfully introduced into natural *XynB*. The maximum activity of xylanase (optiXynB) secreted by *P. pastoris* pPICZaA-optiXynB was 1299 U/mL after 96 h induction. Purified recombinant optiXynB had the molecular weight of 24 kDa. The optiXynB presented highest activity in pH 5.0 and 50 °C. The recombinase was highly specific towards birchwood xylan, beechwood xylan, and oat-spelt xylan. In the broiler experiment, a total of 200 Arbor Acre broilers (one day old) were randomly allocated into four groups fed with basal diets containing 0 (control group), 500, 1000, and 1500 IU/kg optiXynB. Dietary 1000 and 1500 IU/kg optiXynB significantly increased (*p* < 0.05) final weight and body weight gain; dietary 500, 1000, and 1500 IU/kg optiXynB significantly increased (*p* < 0.05) pre-evisceration weight, dressed percentage, and eviscerated weight compared with the control group. Inclusion of optiXynB in broiler diets linearly increased final weight, body weight gain, breast muscle weight and leg muscle weight, but linearly decreased feed conversion rate (*p* < 0.05). Furthermore, inclusion of optiXynB in broiler diets linearly and quadratically increased pre-evisceration weight, dressed percentage, and eviscerated weight (*p* < 0.05). The recombinant optiXynB from *P. pastoris* pPICZaA-optiXynB was beneficial in improving growth performance and carcass characteristics of broilers.

## 1. Introduction

Xylan, the substrate for xylanase, is one of the important anti-nutritional factors in plant feed, including corn, soybean, triticale, and wheat [1,2]. Endo-β-1,4-xylanase (XynB), belonging to glycoside hydrolases family 10 and 11, can efficiently degrade β-1,4-xylosidic bonds of xylan into different length xylooligosaccharides [3]. Several reports document that the xylanase can reduce intestinal viscosity, increase the digestibility of nutrients, and improve the performance of broilers through degradation of xylan [4,5]. Therefore, XynB is an important feed enzyme, and its demand has increased rapidly in broiler feed production.

Although xylanases generally exist in different sources, microorganisms are still the main resource for production of xylanases. The fungus *Trichoderma reesei* is considered as an efficient xylanase and glucanase producer [6]. However, the total activity of beta-xylanase from *T. reesei* Rut C−30 was not commercially feasible [7]. In addition, the culture supernatant of *T. reesei* includes many undesirable enzymes, which may be a problem for some applications [7]. To solve these problems, heterologous expression and codon optimization technology were used to produce higher yield and more purity xylanase.

The *Pichia* system has been widely applied in expression of foreign enzymes, including phytase, glucanase, amylase, and mannanase [8,9]. However, achieving the maximum production yield of foreign enzyme is much more difficult due to the codon biases differences between the *P. pastoris* and the wild strain [10]. Codon biases may decrease the diversity of isoacceptor tRNAs and enhance the expression level of foreign genes in a *P. pastoris* expression system [10,11]. Specifically, rare codons that exist in clusters will greatly decrease the expression level of recombinant proteins in a host [10,12]. The codon optimization technique thus has been developed to increase expression of recombinant proteins.

There are few systematic studies including improvement of xylanase yield and its effect on the growth performance of broiler. In the present work, the natural *XynB* from *T. reesei* was optimized according to preferred codons for achieving a high expression level of xylanase in *P. pastoris*. The optimized xylanase (*optiXynB*) gene was synthesized by whole gene assembly and expressed in the *P. pastoris*. The biochemical characterizations of the optiXynB were determined. Furthermore, the effects of recombinase on growth performance and carcass characteristics were also studied in broiler production. The aim of this work is to combine optiXynB expression and broiler production. 

## 2. Materials and Methods

The broiler experiment was performed according to the guidelines for the care and use of experimental animals of the Ministry of Science and Technology of the People’s Republic of China (Approval Number 2006-398) and approved by the College of Animal Science, Anhui Science and Technology University.

### 2.1. Strains, Vectors, Reagents, and Culture Medium

*Escherichia coli* DH5α was purchased from TIANGEN (Beijing, China). *P. pastoris* X-33 and expression plasmid pPICZαA were conserved at −80 °C. *EcoR*І, *Kpn*І, *Sac*I, and universal DNA purification kits were purchased from TaKaRa (Beijing, China). Buffered glycerol-complex medium (BMGY), buffered methanol-complex medium (BMMY), and yeast extract peptone dextrose (YPD) were prepared based on the protocols provided by Invitrogen (Carlsbad, CA, USA).

### 2.2. Design and Synthesis of the OptiXynB Gene 

According to the usage frequency of codons in *P. pastoris*, the *T. reesei* xylanase gene, without a native signal peptide (GenBank: EU532196), was optimized by replacing the preferred codons without changing the encoded amino acids. The *optiXynB* gene, with *EcoR*І and *Kpn*І restriction sites added at its 5´ and 3´ ends, was synthesized by Geneary Biotech Co., Ltd. (Shanghai, China). Then, the *optiXynB* gene was cloned into the expression vector pPICZαA in *E. coli* DH5α.

### 2.3. Transformation into P. pastoris

The expression plasmid, pPICZαA-optiXynB, was linearized by *Sac*I and then transformed into *P. pastoris* X-33 using the electroporation method in an Eppendorf Multiporator (Germany) based on its manufacturer’s instruction. The positive clones were selected and placed on YPDZ-plates (100 μg/mL zeocin) at 29 °C for 2–4 days. The recombinant clones were tentatively identified by colony PCR and DNA sequencing analysis by TaKaRa (Beijing, China).

### 2.4. Expression of optiXynB

The selected transformants of *P. pastoris* pPICZαA-XynB were grown in 50 mL BMGY at 29 °C and 250× *g* until the OD_600_ reached a peak value between 4 and 6. The cells were gathered by centrifuging at 4800× *g* for 6 min and were resuspended in 25 mL BMMY. Absolute methanol was added at a final concentration of 0.5 % every 12 h to maintain induction. This supernatant was collected daily and stored at −70 °C.

### 2.5. Purification of Recombinant optiXynB

The culture supernatant from *P. pastoris* pPICZαA-XynB was purified using Ni^2+^-chelating chromatography (Cwbiotech, China) based on the manufacturer’s protocol. SDS-PAGE was used to detect the molecular weight of the purified optiXynB. 

### 2.6. Detection of optiXynB Activity

Xylanase activity was measured using the substrate xylan (Sigma-Aldrich, St. Louis, MO, USA). The diluted recombinant protein (0.1 mL) was incubated with 1% xylan (0.9 mL) at 50 °C for 10 min. One international unit (U) of activity was equivalent to the amount of xylanase that caused an increase of 1 μmol reducing sugar in the reaction system. 

### 2.7. Analysis of optiXynB Properties

To determine the optimum pH, 0.05 M buffers including citrate buffer (pH 3.0), citrate phosphate buffer with a pH ranging from 4.0 to 7.0, and phosphate (pH 8.0) were used. In order to detect the optimum temperature for the enzyme, xylanase activity was examined at intervals from 20 to 90 °C (pH 5.0). 

The pH stability of optiXynB was determined by incubating protease in 0.05 M buffer (pH 3.0–8.0) for 30 min at 50 °C. To examine temperature stability, the recombinase was incubated at different temperatures (50, 60, and 70 °C) for 30 min. Then, the relative activity was measured at the optimal temperature and pH values for 10 min.

### 2.8. Analysis of Substrate Specificity

Substrate specificity of optiXynB was measured by different substrates. The reaction was performed in 0.05 M citrate phosphate buffer (pH = 5.0) containing 1% oat-spelt xylan, birchwood xylan, beechwood xylan, sodium carboxymethylcellulose (CMC-Na), and microcrystalline cellulose (MCC), respectively.

### 2.9. Broilers, Management, and Diets

Two hundred Arbor Acre broilers (1 day old) were randomly allocated into 4 groups (5 replicates of 10 broilers per group). The 4 groups were fed with basal diets containing 0 (control group), 500, 1000, and 1500 IU/kg optiXynB. The compositions of diets (shown in Supplementary Table S1) were formulated based on National Research Council (NRC) [13] and Wen et al. [14]. Chickens had ad libitum access to food and fresh water.

### 2.10. Growth Performance

Body weight and feed intake were detected weekly and daily, respectively. The feed conversion ratio was then calculated.

### 2.11. Carcass Characteristics

Fifteen chickens per group (3 chickens per replicate) were selected for sampling. The carcass characteristics, including pre-evisceration weight, dressed percentage, eviscerated weight, percentage of eviscerated yield, breast muscle weight, percentage of breast muscle yield, leg muscle weight, and percentage of leg muscle yield, were detected according to the “Performance terms and measurements for poultry (NY/T 823–2004)”.

### 2.12. Statistical Analysis

Excel 2010 software (Microsoft Corp., Redmond, WA, USA) was used to analyze the optiXynB activity and properties (pH and temperature).

For a broiler experiment, the statistical analysis of data was carried out by one-way ANOVA in SPSS 18.0 software (IBM, Armonk, NY, USA). For all groups, differences were compared by Duncan’s test. Linear and quadratic contrasts were analyzed for the effects of different optiXynB supplementation levels (0, 500, 1000, and 1500 IU/kg) by polynomial contrasts in SPSS 18.0 software. *p* < 0.05 was regarded as significant.

## 3. Results

### 3.1. Design of optiXynB Gene

**Figure 1:**
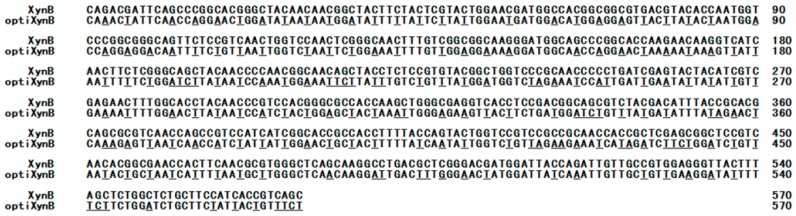
Comparison of *T. reesei* xylanase gene (*XynB*) and optimized xylanase gene (*optiXynB*). The underline letters are the changed codons.

Analysis of the *T. reesei XynB* gene showed that there were various rare codons, including GCG (Ala), CTC (Leu), AGC (Ser), and CGC (Arg) in *P. pastoris* (Figure 1). For high yields of recombinant xylanase, the whole gene synthesis strategy was used to replace the least frequently used codons with more frequently used synonymous codons of *P. pastoris*. Results indicated that about 180 of the rare codons were optimized according the codon bias of the *Pichia* genome (Figure 1). However, they have the same amino acid sequences. The optimized *XynB* gene (*optiXynB*) showed 67.4% identity with the native *XynB* gene (Figure 1). After optimization, the minimal free energy of *optiXynB* was raised from –195.6 to –118.5 kcal/mol.

### 3.2. Expression of optiXynB Gene in P. pastoris

**Figure 2:**
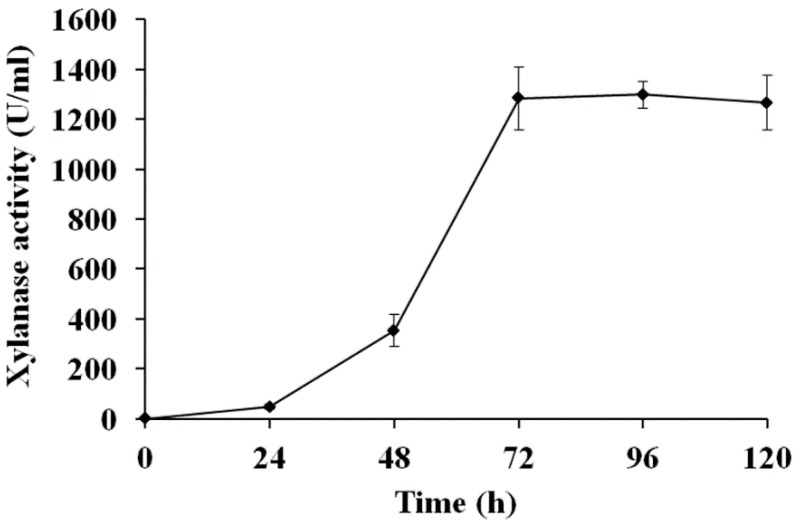
Recombinant xylanase production by *P. pastoris* pPICZαA-optiXynB.

The single positive transformant was selected and induced by methanol. The xylanolytic production was determined every 24 h (Figure 2). After 96 h induction, the highest activity of optiXynB secreted by *P. pastoris* pPICZaA-optiXynB was 1299 U/mL (Figure 2).

### 3.3. SDS-PAGE Determination of Crude and Purified optiXynB

**Figure 3:**
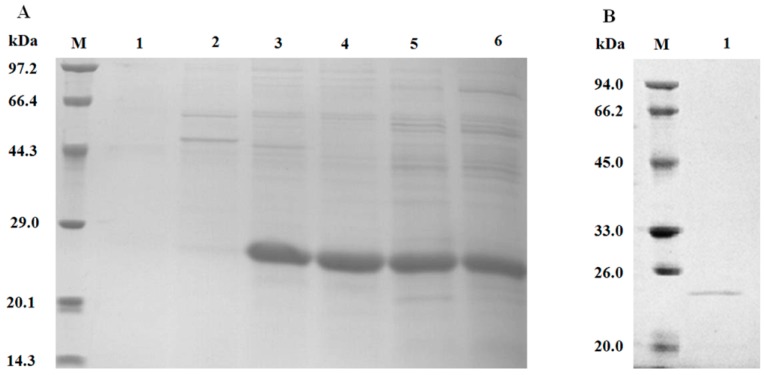
SDS-PAGE analysis. (**A**) Expressed proteins. Lane M: protein MW markers; Lane 1–6: the crude xylanase from *P. pastoris* pPICZαA-optiXynB at 24 h, 48 h, 72 h, 96 h, 120 h, and 144 h. (**B**) Purified optiXynB. Lane M: protein MW markers; Lane 1: the purified optiXynB.

The molecular weight (MW) of recombinant optiXynB was approximately 24 kDa (Figure 3). Figure 3A showed that the optiXynB was the major protein of secreted protein (about 80–90%), as measured using densitometer software in the induced culture medium. After purification of crude protein, one band with a MW of 24 kDa was presented on gel (Figure 3B).

### 3.4. The optiXynB Properties

**Figure 4:**
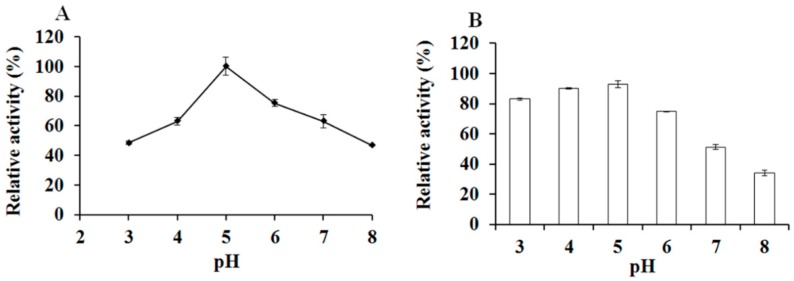
Effect of pH on the optiXynB activity (**A**) and its pH stability (**B**). The maximum value was set as 100 %.

Xylanolytic detection at different pH buffers showed that the optimal pH of the optiXynB was 5.0 (Figure 4A). The activity of optiXynB exhibited less activity (46–48%) at pH values below 3.0 and above 8.0 (Figure 4A). The optiXynB was stable (over 80%) at pH 3–5, and its activity decreased rapidly when incubated at pH 8.0 for 30 min (Figure 4B).

**Figure 5:**
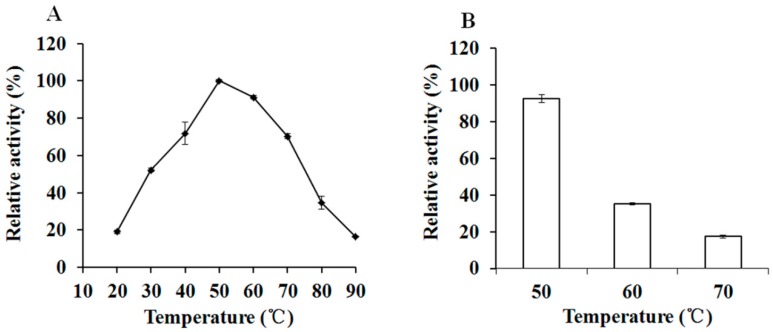
Effect of temperature on the optiXynB activity (**A**) and its temperature stability (**B**). The maximum value was set as 100 %.

The optiXynB was optimal at 50 °C (Figure 5A). The activity of optiXynB showed less activity at temperatures below 20 °C and above 80 °C (Figure 5A). It was stable at 50 °C for 30 min, and kept about 91% of its highest activity (Figure 5B). However, the activity of optiXynB decreased rapidly when incubated at 60 °C and 70 °C for 30 min (Figure 5B).

**Figure 6:**
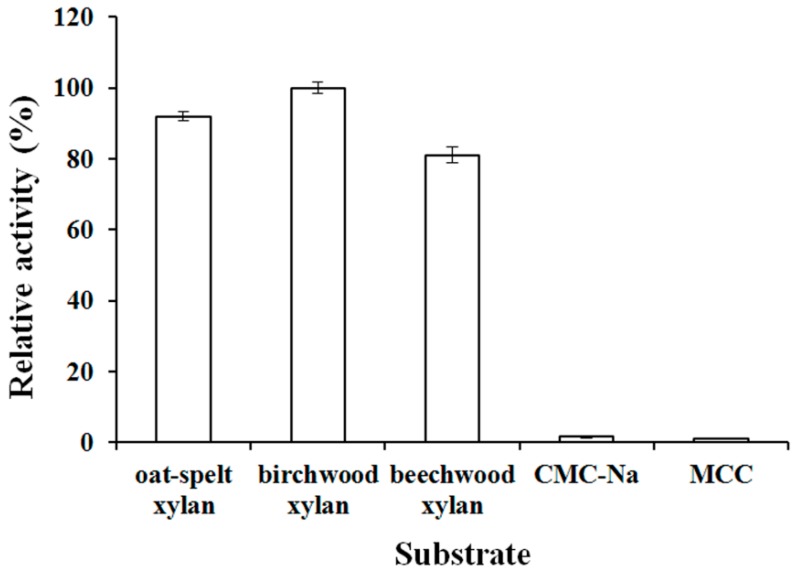
Substrate specificity for the recombinant xylanase. The activity value for birchwood xylan was set as 100%. CMC-Na: sodium carboxymethylcellulose, MCC: microcrystalline cellulose

The activity of optiXynB on hemicellulose and cellulose was determined (Figure 6). It was could efficiently degrade birchwood xylan (100%), beechwood xylan (81%), and oat-spelt xylan (92%). However, the optiXynB was nearly free of cellulolytic activity. 

### 3.5. Growth Performance of Broilers

The effect of optiXynB on the growth performance of broilers is shown in the Table 1. Compared with control group, dietary 1000 and 1500 IU/kg optiXynB significantly increased (*p* < 0.05) final weight and body weight gain. Inclusion of optiXynB into broiler diets linearly increased final weight and body weight gain, but linearly decreased feed conversion rate (*p* < 0.05). However, no significant differences were found in feed intake and feed efficiency between the control and optiXynB group.

### 3.6. Carcass Characteristics of Broilers 

The effect of optiXynB on carcass characteristics of broilers is shown in the Table 2. Compared with control group, dietary 500, 1000, and 1500 IU/kg optiXynB significantly increased (*p* < 0.05) pre-evisceration weight, dressed percentage, and eviscerated weight. Inclusion of optiXynB to broiler diets linearly and quadratically increased pre-evisceration weight, dressed percentage, and eviscerated weight (*p* < 0.05). Furthermore, inclusion of optiXynB into broiler diets linearly increased breast muscle weight and leg muscle weight (*p* < 0.05).

## 4. Discussion

Recently, xylanase has been widely applied to broiler and feed production [15]. *T. reesei* produces xylanase suitable for commercial utilization, but the low production level and other undesirable proteases limit its applications [16]. The production of xylanase using *P. pastoris* systems is an available technology because of high protein yield and purity [17,18]. Many reports have suggested that foreign protein expression levels in recombinant *P. pastoris* are greatly influenced by the differential codon preference between the expressed host and native gene sequence [19]. Consequently, the codon optimizing strategy has been used to increase heterologous protein yield, most often leading to overexpression of the target protein [20].

Comparing the codon usages in *Pichia*, we found many rare codons in the nature xylanase gene from *T. reesei*. The codons for the amino acids, Ser (TCG and AGC), Pro (CCG), Leu (CTC), Ala (GCG), and Arg (CGC) in *T. reesei*, were very infrequently used codons in the *P. pastoris* genome, and this difference could be expected to decrease its expression yields in a *Pichia* system. Yu et al. found that the yield of recombinant protein was correlated with codon usage and the abundance of tRNA isoacceptors in a host [21]. Hu et al. and Ata et al. suggested that the optimizing strategy (exchanging rare codons for ones that are more common) could increase the expression level of recombinant enzyme in *P. pastoris* [10,22]. In this study, the nature xylanase gene codons were successfully optimized based on the *Pichia* genome and synthesized by whole gene assembly. Meanwhile, the minimal free energy of the *optiXynB* gene was considered to increase after optimization, suggesting reduced complexity of mRNA secondary structure. 

The *optiXynB* gene was inserted between a strong *AOX1* promoter and transcription terminator on vector pPICZαA. SDS-PAGE showed that the molecular weight of purified optiXynB was about 24 kDa, which is similar to previous studies [16,23]. This indicated that *optiXynB* was successfully expressed and secreted by recombinant *P. pastoris*. The xylanase activity produced by *P. pastoris* pPICZαA-optiXynB was higher than the xylanases from *T. reesei*, *S. cerevisiae* Y294, *P. pastoris* pPICZαA-XynB, and so on [16,23,24,25]. Moreover, the recombinant optiXynB was also the major protein in the induced culture medium (Figure 3).

The xylanolytic properties of recombinant optiXynB were similar to other xylanases [7,23]. It was active at pH 4.5–5.5, with an optimal pH of 5.0, which is identical to the reported xylanase [7]. Moreover, the optimal temperature of the recombinant xylanase was 50 °C, which is almost the same as that shown in the previous studies [7]. The xylanase was fairly stable at the pH (3–5) and optimum temperature, but was rapidly deactivated with exposure to temperatures of 60–70 °C for 0.5 h.

The XynB for *T. reesei* is classified into glycoside hydrolases family 11 and has five subsites for binding xylopyranose rings [26]. Only few XynB (glycoside hydrolases family 11) were found to have binding sites of cellulose [27]. Similar results were also found in optiXynB. The optiXynB showed high activities for different xylan sources, while it exhibited low activity for cellulose. Based on the broad substrate specificity, the effects of optiXynB on broiler growth performance and carcass characteristics were explored in the present study.

Corn and soybean meal is the most commonly used in the feed diet of poultry. Xylan, which exists in the corn and soybean meal, cannot be hydrolyzed by poultry endogenous enzymes [4,28]. The anti-nutritional effect of feed xylan is reduction of nutrient digestibility and growth performance in broiler production [28]. It is well known that the xylanase can effectively degrade xylan encapsulated in the starch and protein of plant feed, which decrease the barriers to nutrients digestion and utilization [4,5,29]. This work indicated that the supplement of optiXynB increased final weight and body weight gain of broilers in comparison with control group. There were significant linear effects of dietary optiXynB in final weight, body weight gain, and feed conversion rate of broilers. Similar results were also found in the report of Cowieson and Nian et al., where the growth performance was improved for broilers fed corn-based diets with xylanase [30,31].

Poultry carcass characteristics are one of the main factors that affect the production rate and commercial value of meat [32]. Compared with the control group, dietary optiXynB increased pre-evisceration weight, dressed percentage, and eviscerated weight. Furthermore, the linear and quadratic effects in the pre-evisceration weight, dressed percentage, and eviscerated weight were found with increasing optiXynB supplementation. The improvements of carcass characteristics were caused by the better growth performance in the optiXynB group. Chen et al. suggested that the addition of xylanase could significantly improve carcass traits of Guangxi partridge broilers [33]. These findings suggested that opitiXynB had positive effects on broiler production.

## 5. Conclusions

The objective of the present study was to increase expression level of *T. reesei* xylanase in *P. pastoris* and explore its application in the broiler production. The yield of optiXynB was greatly improved by codon optimization strategy. The molecular weight of recombinant optiXynB was approximately 24 kDa. The optimal pH and temperature of the optiXynB were 5.0 and 50 °C, respectively. In the broiler experiment, dietary optiXynB improves the growth performance and carcass characteristics of broilers. The present study not only provides a valuable strategy to increase expression level of xylanase in *P. pastoris*, but also provides an available approach to increase production performance of broilers fed corn-soybean feed. Future work in our laboratory will focused on comparisons between optiXynB and other types of xylanases in broiler production.

## Figures and Tables

**Table 1:** Effects of dietary optiXynB on growth performance of broilers.

Item	Treatment				SEM ^1^	*p*-Value	
	Control	500 IU/kg	1000 IU/kg	1500 IU/kg		Linear ^2^	Quadratic ^2^
Final weight (g)	2022.8a	2036.9ab	2047.1b	2055.9b	4.26	0.003	0.711
Body weight gain (g)	1975.9a	1990.2ab	1999.8b	2009.3b	4.31	0.003	0.735
Feed intake (g)	4340.8	4314.5	4315.3	4305.2	15.82	0.494	0.813
Feed conversion rate (g/g)	2.20	2.17	2.16	2.14	0.01	0.033	0.667

a, b: Values in rows with different letters differ significantly (*p* < 0.05). *n* = 50 broilers. ^1^ SEM: standard error of the mean. ^2^ Linear and quadratic contrasts were analyzed for the effects of different optiXynB supplementation levels by polynomial contrasts in SPSS 18.0 software.

**Table 2:** Effects of dietary optiXynB on carcass characteristics of broilers.

Item	Treatment				SEM ^1^	*p*-Value	
	Control	500 IU/kg	1000 IU/kg	1500 IU/kg		Linear ^2^	Quadratic ^2^
Pre-evisceration weight (g)	1801.8a	1831.1b	1849.8c	1846.2c	4.88	<0.001	0.004
Dressed percentage (%)	89.2a	90.0b	90.5b	90.1b	0.15	0.006	0.021
Eviscerated weight (g)	1339.5a	1351.4b	1352.8b	1353.9b	1.64	<0.001	0.019
Percentage of eviscerated yield (%)	66.3	66.4	66.2	66.1	0.12	0.397	0.727
Breast muscle weight	331.8	343.6	348.9	349.9	3.24	0.043	0.385
Percentage of breast muscle yield (%)	24.8	25.4	25.8	25.8	0.23	0.101	0.533
Leg muscle weight	286.8	289.9	295.2	299.4	2.35	0.049	0.903
Percentage of leg muscle yield (%)	21.4	21.5	21.8	22.1	0.16	0.109	0.675

a, b: Values in rows with different letters differ significantly (*p* < 0.05). *n* = 15 broilers. ^1^ SEM: standard error of the mean. ^2^ Linear and quadratic contrasts were analyzed for the effects of different optiXynB supplementation levels by polynomial contrasts in SPSS 18.0 software.

## Supplementary Materials

The following are available online at http://www.mdpi.com/2076-2615/9/2/65/s1; Table S1. Ingredient and composition of basal diets (g/kg).
