# Supplementary material for: Efficient Expression of Xylanase by Codon Optimization and Its Effects on the Growth Performance and Carcass Characteristics of Broiler
Source: Animals (Basel). 2019 Feb 20;9(2):65. doi: 10.3390/ani9020065 (PMC6406647; doi:10.3390/ani9020065)
Supplement: Supplementary file 1 [file animals-09-00065-s001.pdf]

animals

# Supplementary Materials: Efficient Expression of Xylanase by Codon Optimization and Its Effects on the Growth Performance and Carcass Characteristics of Broiler

**Hong Hu [†], Sifa Dai [†], Aiyou Wen and Xi Bai ***

College of Animal Science, Anhui Science and Technology University, Fengyang, 233100, China; haiyanghh@163.com (H.H.); daisifa-004@163.com (S.D.); aywen2008@126.com (A.W.)

* Correspondence: baixi1987326@163.com; Tel.: +86-0550-6732040

† These authors contributed equally to the experimentations.

Received: 13 January 2019; Accepted: 16 February 2019; Published

**Supplementary Table 1.** Ingredient and composition of basal diets (g/kg).

| | Starter diet (1–21 d) | Grower diet (22–42 d) |
|---|---|---|
| **Dietary ingredients (g/kg)** | | |
| Ground yellow maize | 514.1 | 566.5 |
| Maize starch | 10.0 | 10.0 |
| Soybean meal | 386.5 | 341.0 |
| Fish meal | 35.0 | 20.0 |
| Soybean oil | 30.0 | 35.0 |
| Dicalcium phosphate | 9.5 | 11.0 |
| Ground limestone | 9.0 | 10.5 |
| Iodized salt | 3.0 | 3.0 |
| DL-Methionine | 0.9 | 1.0 |
| Micronutrients [a] | 2.0 | 2.0 |
| **Composition calculated (g/kg)** | | |
| AME (MJ/kg) | 11.35 | 12.46 |
| Lysine | 12.5 | 11.4 |
| Methionine | 4.8 | 4.3 |
| Methionine + cystine | 8.4 | 7.6 |
| Non-phytate phosphorus | 4.2 | 3.7 |
| **Analyzed (g/kg)** | | |
| Crude protein | 231.2 | 204.7 |
| Calcium [c] | 10.3 | 8.8 |

[a] Provided per kilogram of diet: vitamin A (as all-*trans* retinol acetate), 10,000 IU; cholecalciferol, 2600 IU; vitamin $B_6$, 3.0 mg; vitamin $B_{12}$, 0.014 mg; thiamin (as thiamin mononitrate), 1.6 mg; vitamin E (as all-rac-$\alpha$-tocopherol acetate), 20 IU; vitamin K (as menadione sodium bisulfate), 2.0 mg; riboflavin, 6.0 mg; calcium pantothenate, 20 mg; niacin, 30 mg; folic acid, 0.8 mg; biotin, 0.12 mg; choline (as choline chloride), 500 mg; iron (from iron sulfate), 80 mg; iodine (from potassium iodide), 0.35 mg; selenium (from sodium selenite), 0.15 mg; zinc (from zinc sulfate), 40 mg; copper (from copper sulfate), 8 mg.
